# HCV Phylogeography of the General Population and High-Risk Groups in Cyprus Identifies the Island as a Global Sink for and Source of Infection

**DOI:** 10.1038/s41598-019-46552-7

**Published:** 2019-07-11

**Authors:** Dimitrios Paraskevis, Dora C. Stylianou, Johana Hezka, Zachariah Stern, Martha Oikonomopoulou, Ioannis Mamais, Cyprus PWID Network, Chrysanthos Georgiou, Natasa Savvopoulou, Kyriakos L. Veresies, Tina Pavlou, Argyris Argyriou, Elena Zarouna, Leondios G. Kostrikis

**Affiliations:** 1 Department of Hygiene, Epidemiology and Medical Statistics, Medical School, National and Kapodistrian University of Athens, Athens, Greece; 2 Department of Biological Sciences, University of Cyprus, Nicosia, Cyprus; 3 Cyprus National Addictions Authority, Nicosia, Cyprus; 4 Veresie Clinic, Larnaca, Cyprus; 5 Therapeutic Community Aghia Skepi, Nicosia, Cyprus; 6 Gephyra Substitution Treatment Programme, Nicosia, Cyprus; 7 Anosis Detoxification Unit and Sosivio Substitution Treatment Programme, Limassol, Cyprus; 8 Ithaki Counseling Center, Nicosia, Cyprus

**Keywords:** Hepatitis C, Epidemiology

## Abstract

Hepatitis C virus (HCV) genotype and subtype distribution differs according to geographic origin and transmission risk category. Previous molecular epidemiology studies suggest the presence of multiple subtypes among Cypriot subjects. To investigate HCV genotype- and subtype-specific dissemination patterns, origins, and transmission in Cyprus, we analyzed HCV sequences encoding partial Core-E1 and NS5B regions. Analyzed populations comprised the general population and high-risk cohorts in Cyprus and a globally sampled dataset. Maximum-likelihood phylogeny reconstruction with bootstrap evaluation, character reconstruction using parsimony, and bootstrap trees estimated by ML were performed to identify the geographic origin of HCV subtypes and statistically significant dispersal pathways among geographic regions. Phylogeographic analyses traced the origin of subtypes in the general population and among PWID in Cyprus to unique and overlapping globally distributed regions. Phylogenetic analysis in Core-E1 revealed that most sequences from incarcerated populations in Cyprus clustered with the general population and PWID. We estimate that HCV infections in Cyprus originate from multiple global sources while most HCV transmissions among incarcerated individuals occur locally. This analysis is one of a few studies tracing HCV dispersal patterns using global datasets, and these practices and findings should inform how HCV epidemics are targeted by future prevention policies.

## Introduction

Hepatitis C virus (HCV) is a major cause of chronic liver disease, cirrhosis, and hepatocellular carcinoma, giving rise to a major public health issue on a global scale^1^. It is a highly genetically diverse single-stranded positive-sense RNA virus in the *Flaviviridae* family^2^. The prevalence of Hepatitis C virus globally has been estimated to be approximately 180 million infections. HCV is phylogenetically classified into 7 major genotypes (clades) and 57 subtypes (subclades)^3^. Geographic origin and transmission risk category influence the global distribution of HCV genotypes and subtypes^4^.

Previous estimates reported that genotype 1 was the most prevalent (46.2%), followed by 3a (30.1%)^5^. Genotypes 2, 4, and 6 account for most of the remaining infections with prevalences estimated at 9.1%, 8.3%, and 5.4%, respectively^5^. Genotype 5 was estimated at <1%. Genotypes 1 and 3 have been found globally, whereas genotypes 4 and 5 occur mostly in low-income countries^5^. Genotype 1 circulates at highest prevalence in the Americas, Central Europe, and a few countries in Asia; genotype 2 in Western Africa; genotype 3 in Central Asia, Latin America, and Eastern Europe; genotype 4 in North Africa, Central Africa, and the Middle East; genotype 5 in Southern Africa; and genotype 6 in Eastern and Southeast Asia^5^. However, the origin of HCV and the global patterns of HCV dissemination remain unknown.

Our previous molecular epidemiology studies suggested that among Cypriot subjects, 1b and 3a were the most predominant subtypes, followed by 1a. Specifically, 3a was the most prevalent (57%) among people who inject drugs (PWID) and 1b among the general population. For people who inject drugs, the 2k/1b recombinant type that has been previously described in Russia was also detected^6^.The global distribution of subtypes 1a and 1b has likely been shaped by the dissemination of blood and blood products after the Second World War^7^, while the distribution of genotype 3 is likely a result of the mobility of PWID and drug trafficking^8–10^. In Cyprus, HCV infection from intravenous drug use is associated with each prevalent subtype^11^. With these transmission mechanisms, local and global human movement primarily drive the phylogeography of HCV due to the evolutionary dynamics of the virus^12^.

Our objective was to investigate the dissemination and transmission patterns of the common Hepatitis C virus subtypes in Cyprus using phylogeographic analysis. This study is an examination of how subtype and geographic distribution affect the transmission patterns of HCV into, out of, and within Cyprus and are informed by certain risk factors. Phylogeographic analysis, which has been underutilized to studies of trends of HCV distribution and dissemination patterns, is an applicable tool to assess these topics.

## Methods

### HCV sequences

As part of this study, newly-obtained HCV sequences of both the Core-E1 region (21 sequences, 417 nucleotides-long corresponding to positions 867–1283 of the reference sequence of the H77 strain (accession number AF009606)) and NS5B region (20 sequences, 405-nucleotide-long, positions 8277–8681) were obtained in a prospective HCV molecular epidemiology study (2011–2012) of intravenous drug-users attending therapy in Cyprus. Blood samples were collected from 67 consenting study subjects in accordance with relevant guidelines and regulations by and approval from the National Bioethics Committee in Cyprus (EEBK/EΠ/2011/07) and all samples were processed as previously described^13^. An informed consent has been obtained from each participating study subject. The newly obtained sequences were submitted to GenBank (accession numbers MK682535-MK682555 for the Core-E1 region and MK682556-MK682575 for the NS5B region). Additionally, HCV sequences (N = 221) from the general population and high-risk cohorts from our previously published HCV molecular epidemiological studies in Cyprus were also used in the phylogeographic analyses^11,13,14^ as follows: 14 (1a), 53 (1b) and 44 (3a) of the Core-E1 region and 11 (1a), 54 (1b) and 45 (3a) of the NS5B region.

Multiple sequences from the same patient and duplicates (100% identical) were excluded from the alignments. Duplicates were detected using the ElimDupes online tool, available at Los Alamos HIV sequence database [https://www.hiv.lanl.gov/content/sequence/elimdupesv2/elimdupes.html]. Sequences from unknown country of origin were also omitted from the analyses. The genotypes of all HCV sequences were confirmed using the COMET-HCV automated subtyping tool (https://comet.lih.lu/). All sequences falsely assigned to subtypes 1a, 1b, or 3a were discarded from the alignment. Sequence alignments and editing were carried out manually in line with the encoded reading frame using the MEGA6 software^15^. The final alignments in Core-E1 and NS5B were of 417 and 405 nucleotides in length, respectively. The number of sequences analyzed were 1,200 (1a), 1,289 (1b), and 318 (3a) in Core-E1 and 1,052 (1a), 967 (1b), and 346 (3a) in NS5B. A random down sampling was performed for the alignments of 1a in both Core-E1 and NS5B regions at 5%, 10%, and 20% of United States sequences since it is the geographic area with the largest representation in the database and therefore in our analysis. The down sampling was performed to assess the impact of sampling from the area with the largest representation on the estimation of viral migration.

### Phylogenetic analyses

Maximum-likelihood phylogeny reconstruction with bootstrap evaluation was conducted in RAxML-HPC Blackbox interface version 8.2.4^16^, using the GTR nucleotide substitution model and gamma (Γ) distribution of rate variability among sites (GTR + gamma). Tree visualization was implemented using FigTree v1.4.2 [http://tree.bio.ed.ac.uk/software/figtree/]^17^.

### Phylogeographic and subgroup analysis

Phylogeographic analysis was used to estimate viral migration events and determine the geographical origin and distribution of HCV infections in and from Cyprus. Migration events were inferred by using a cladistics approach as described by Slatkin and Maddison^18^ as implemented in PAUP* 4.0b10^19^. Geographic location (sampling area) was assigned at the tips of the maximum likelihood inferred phylogenetic trees in partial Core-E1 and NS5B regions, for subtypes 1a, 1b and 3a. Ancestral character states were reconstructed at the tree nodes by parsimony. Migration events corresponded to character changes (geographic locations) across the tree topology. To account for phylogenetic uncertainty, migration events were estimated over all bootstrap trees and were calculated as the median of the distribution inferred from all trees used in the analysis as described previously^20^.

Six migration matrices corresponding to the 2 genomic regions each with 3 subtypes were created. To estimate which viral mobility pathways were statistically higher than expected under the null hypothesis of geographic panmixis (full geographic mixing), a statistical phylogeographic analysis was performed as described previously^20^. The distribution of migration events estimated over the bootstrap trees (observed values) for each genotype in each genomic region was checked against the distribution of events estimated over the same set of trees after a random reshuffling of taxa (expected values) to assess for statistical differences. Reshuffling of taxa was performed on the bootstrap-reconstructed trees, using Mesquite program version 3.5^21^. The distributions of observed and expected migrations events for each migration matrix were compared using the 1- sided Mann-Whitney test. Due to multiple comparisons, the significance level (α = 0.05) was adjusted according to Bonferroni correction.

For all migration events, the ratio of the mean of observed versus mean of expected migration events was estimated, ignoring those with expected migration events with a mean of zero. This ratio provides a measure of the amount of viral flow between different countries or subgroups that is not attributed to chance. For example, a ratio of 0.70 suggests that the observed viral flow across the pathway is 30% lower than what is expected from chance while a ratio of 1.30 suggests a 30% higher viral flow than the expected. Such ratios are sensitive to the mean, as expected events with a mean close to “0” may produce high ratio values.

### Nucleotide Sequence Accession Numbers

The accession numbers for HCV sequences obtained in the molecular epidemiology study (2011–2012) of intravenous drug-users attending therapy in Cyprus and submitted to GenBank are MK682535-MK682555 for the Core-E1 region (21 sequences) and MK682556-MK682575 for the NS5B region (20 sequences). The accession numbers for the sequences will be released by GenBank on December 31, 2019 or upon the publication of the manuscript, whichever occurs first.

GenBank accession numbers for HCV sequences obtained in previously published HCV molecular epidemiological studies in Cyprus and used in this study are: for the general population, EU684661-EU684737^11^ and HQ537010-HQ537012^14^ for the Core-E1 region and EU684591-EU684660^11^ and HQ537033-HQ537036^14^ for the NS5B region; for the incarcerated population, HQ537013-HQ537025 for the Core-E1 region and HQ537037-HQ537052 for the NS5B region^14^; for HIV-1/HCV co-infected individuals, HQ537026-HQ537032 for the Core-E1 region and HQ537053-HQ537059 for the NS5B region^14^; and for previously described HCV strains found among people who inject drugs, GQ332540-GQ332553 for the Core-E1 region and CQ332554-CQ332565 for the NS5B region^13^.

## Results

### Phylogeographic analysis- tracing cross-border HCV transmissions

The patterns of cross-border HCV dispersal for the most prevalent subtypes (1a, 1b, and 3a) in Cyprus were estimated by means of statistical phylogeographic analysis using globally sampled HCV sequences as references. The analysis was performed in two partial genomic regions (Core-E1 and NS5B) for which reference sequences from diverse geographical areas comprising Cyprus and globally distributed countries were available. The availability of global reference sequences allows phylogeographic analysis in partial Core-E1 and NS5B regions to reveal HCV epidemiological relationships between Cyprus and the respectively sampled regions from each area. Inferences about the origin of HCV transmission events were based on estimations of the number of viral mobility events (migration events) between sampled locations.

**Figure 1:**
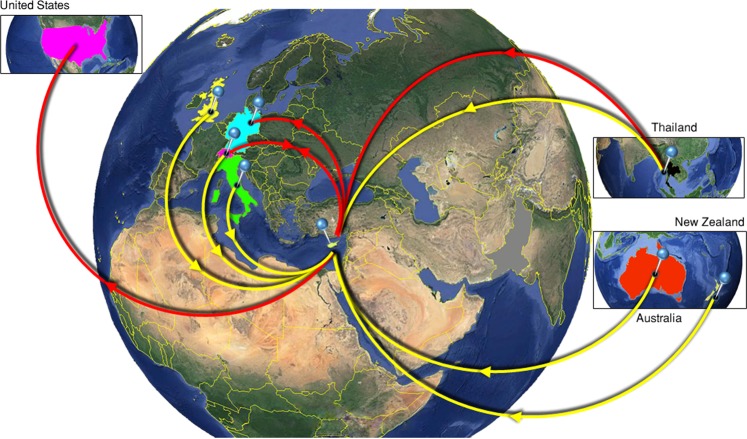
Map of HCV 1a transmission. Geographic origins of HCV subtype 1a infection in Cyprus based on statistical phylogeographic analysis in partial Core-E1 (red lines) and NS5B (yellow lines). Countries acting as “sources” or “sinks” for HCV 1a transmission to Cyprus are highlighted. Arrows indicate direction of HCV 1a mobility. Map images courtesy of Google Earth Pro 7.3.2.5776 (December 14, 2015). Center: Global view centered on Europe. 36°16′38.78″N 36°07′29.71″E, Eye alt 7949.12 km. US Dept of State Geographer, DATA SIO, NOAA, U.S. Navy, NGA, GEBCO. Image Landsat/Copernicus. 2018 © Google. Left: United States of America. 39°14′14.35″N 97°53′40.02″W, Eye alt 7891.99 km. DATA SIO, NOAA, U.S. Navy, NGA, GEBCO. 2019 © GeoBasis-DE/BKG. Upper right: Thailand. 11°37′05.80″N 101°06′23.76″E, Eye alt 8443.62 km. US Dept of State Geographer, DATA SIO, NOAA, U.S. Navy, NGA, GEBCO. Image Landsat/Copernicus. 2018 © Google. Lower right: Australia and New Zealand. 22°19′14.79″S 135°07′28.52″E, Eye alt 7475.94 km. US Dept of State Geographer, DATA SIO, NOAA, U.S. Navy, NGA, GEBCO. Image Landsat/Copernicus. 2018 © Google. https://www.google.com/earth/versions/#earth-pro [April 10, 2019]. The countries of interest here highlighted and arrows were added to indicate the trafficking of the virus based on data shown in the Supplementary Information (Supplementary Tables S1 and S2).

Phylogeographic analysis of Core-E1 revealed that subtype 1a infection in Cyprus originated from Switzerland and Thailand (Supplementary Fig. S1a) while analysis of NS5B revealed infections originating from Switzerland and Thailand, but also Great Britain, Italy, Australia and New Zealand (Fig. 1; Supplementary Fig. S2a). Subsequent statistical phylogeographic analysis revealed the locations of significant sources of HCV viral mobility to Cyprus. The viral mobility events estimated between the different locations are described in Supplementary Tables S1a and S2a. Interestingly, for both genomic regions, the United States of America (USA) was not revealed as a significant source for the 1a subtype in Cyprus (Fig. 1). The estimated viral migrations shown in Supplementary Tables S1a and S2a correspond to population size equal to those used as the source (exporting) and sink populations in this analysis.

Inferences about the trans-continental origin of HCV subtype 1a in Cyprus are likely more credible in NS5B, where both European and non-European references are available. Although Cyprus exported 1a infections to Germany and Switzerland (Core-E1), no outgoing viral mobility was observed within the NS5B region. Based on these patterns of incoming and outgoing viral mobility, Cyprus was received subtype 1a infections and provided limited subsequent dispersal to other locations. The USA was not among the source areas for subtype 1a to Cyprus. To further confirm the inferred pattern for the non-importing nature of the epidemic from the USA, phylogeographic analysis after a random down sampling of references from the USA was repeated. Two sampling rounds down to 20% and 10% of the US sequences were performed while the rest of the dataset was stable. For both analyses, importing viral mobility from the USA to Cyprus was not significant, thus confirming the initial inference about the non-importing pattern for the subtype 1a epidemic in Cyprus.

**Figure 2:**
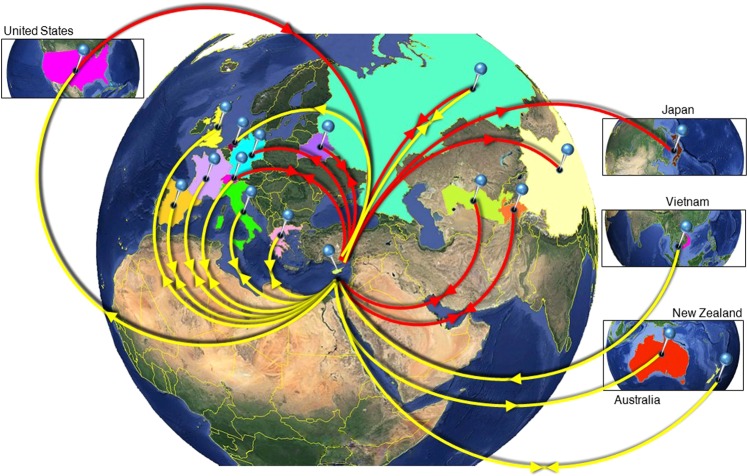
Map of HCV 1b transmission. Geographic origins and destinations of subtype 1b infection in and from Cyprus based on statistical phylogeographic analysis in partial Core-E1 (red lines) and NS5BA (yellow lines). Countries acting as “sources” or “sinks” for HCV 1b transmission to and from Cyprus are highlighted and marked with outgoing and/or incoming arrows indicating the direction of viral mobility. Map images courtesy of Google Earth Pro 7.3.2.5776 (December 14, 2015). Center: Global view centered on Europe. 36°16′38.78″N 36°07′29.71″E, Eye alt 7949.12 km. US Dept of State Geographer, DATA SIO, NOAA, U.S. Navy, NGA, GEBCO. Image Landsat/Copernicus. 2018 © Google. Left: United States of America. 39°14′14.35″N 97°53′40.02″W, Eye alt 7891.99 km. DATA SIO, NOAA, U.S. Navy, NGA, GEBCO. 2019 © GeoBasis-DE/BKG. Upper right: Japan. 36°12′17.37″N 138°15′10.50″E, Eye alt 7471.67 km. US Dept of State Geographer, DATA SIO, NOAA, U.S. Navy, NGA, GEBCO. Image Landsat/Copernicus. 2018 © Google. Middle right: Vietnam. 22°11′45.91″N 110°51′46.67″E, Eye alt 10145.08 km. US Dept of State Geographer, DATA SIO, NOAA, U.S. Navy, NGA, GEBCO. Image Landsat/Copernicus. 2018 © Google. Lower right: Australia and New Zealand. 22°19′14.79″S 135°07′28.52″E, Eye alt 7475.94 km. US Dept of State Geographer, DATA SIO, NOAA, U.S. Navy, NGA, GEBCO. Image Landsat/Copernicus. 2018 © Google. https://www.google.com/earth/versions/#earth-pro [April 10, 2019]. The countries of interest here highlighted and arrows were added to indicate the trafficking of the virus based on data shown in the Supplementary Information (Supplementary Tables S1 and S2).

Similar analysis of the Core-E1 region for HCV subtype 1b, which is the most predominant subtype in the general population in Cyprus, suggested that the sources of origin were Belarus, Russia, Switzerland, Tajikistan, Uzbekistan and the USA (Supplementary Fig. S1b). Analysis of the subtype 1b NS5B region suggested that France, Greece, Italy, Netherlands, New Zealand, Russia, Spain, Switzerland, and Vietnam were the origins of infection within Cyprus (Fig. 2; Supplementary Fig. S2b). Viral migration matrices are shown in Supplementary Tables S1b and 2b. Significant trans-continental mobility (incoming) of subtype 1b was observed from the USA, Oceania, and South Eastern Asia, while Western Europe, Central Asia, and Eastern Europe produced lower levels of incoming mobility. Unlike for subtype 1a, the USA was a significant source of subtype 1b viral infections in Cyprus. Cyprus was also a source for outgoing subtype 1b viral mobility to Belarus, China, Germany, Japan, Russia, Switzerland, Tajikistan, and Uzbekistan (Core-E1) and Australia, France, Greece, Italy, New Zealand, Russia, Spain, Switzerland, Great Britain, and the USA (NS5B) (Fig. 2). Cyprus served both as a global sink and a source for subtype 1b infections in both the Core-E1 and NS5B regions.

**Figure 3:**
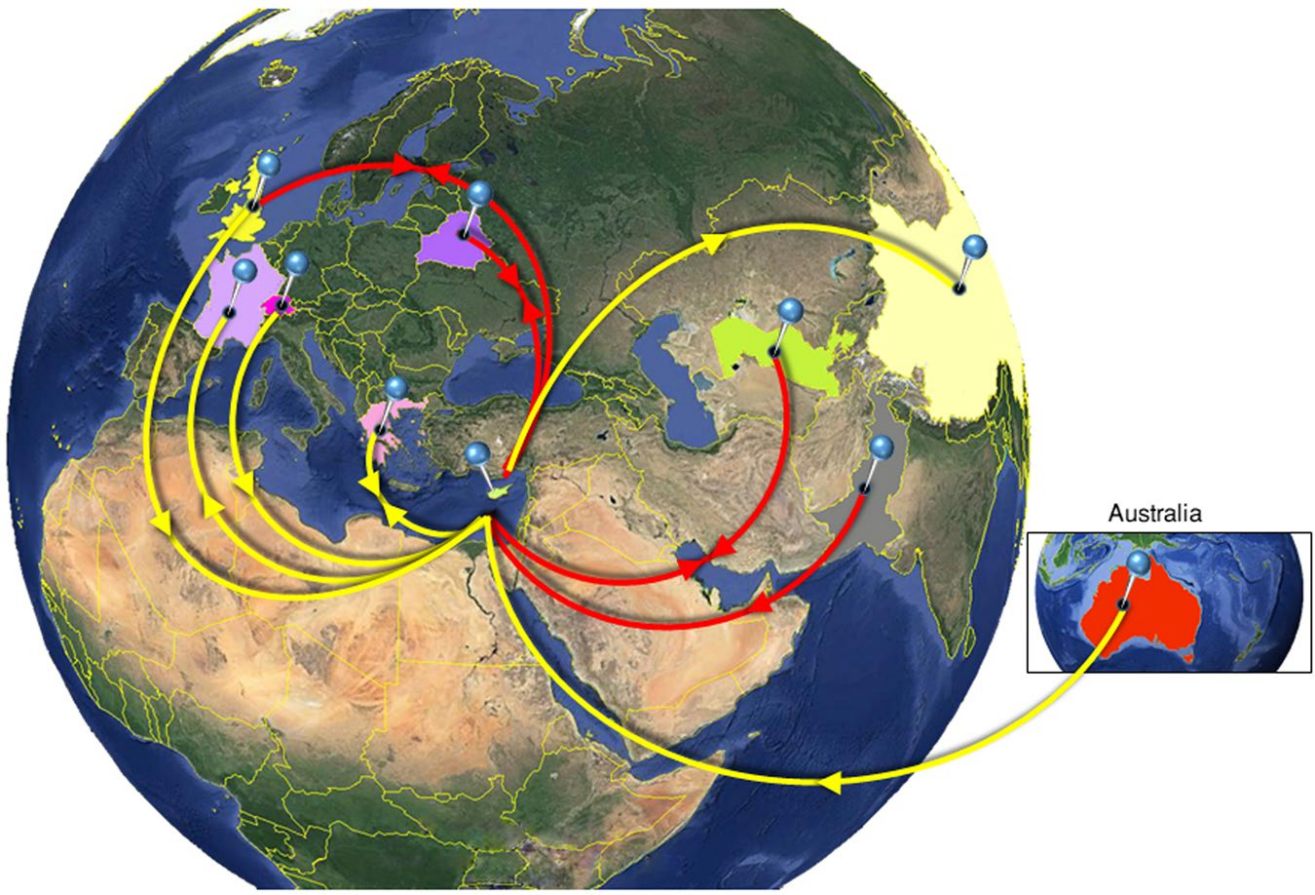
Map of HCV 3a transmission. Geographic origin of subtype 3a infection in Cyprus based on statistical phylogeographic analysis in partial Core-E1 (red lines) and NS5BA (yellow lines). Countries acting as “sources” or “sinks” for HCV 3a transmission to and from Cyprus are highlighted and marked with outgoing and/or incoming arrows indicating the direction of viral mobility. Map images courtesy of Google Earth Pro 7.3.2.5776 (December 14, 2015). Center: Global view centered on Europe. 36°16′38.78″N 36°07′29.71″E, Eye alt 7949.12 km. US Dept of State Geographer, DATA SIO, NOAA, U.S. Navy, NGA, GEBCO. Image Landsat/Copernicus. 2018 © Google. Right: Australia. 22°19′14.79″S 135°07′28.52″E, Eye alt 7475.94 km. US Dept of State Geographer, DATA SIO, NOAA, U.S. Navy, NGA, GEBCO. Image Landsat/Copernicus. 2018 © Google. https://www.google.com/earth/versions/#earth-pro [April 10, 2019]. The countries of interest here highlighted and arrows were added to indicate the trafficking of the virus based on data shown in the Supplementary Information (Supplementary Tables S1 and S2).

Finally, HCV subtype 3a, which is predominant among PWID, originated from Belarus, Pakistan, Great Britain, and Uzbekistan (Core-E1) (Supplementary Fig. S1c), Switzerland, Greece, Great Britain, and Australia (NS5B) (Fig. 3; Supplementary Fig. S2c). Analysis revealed that 3a infections were introduced to Cyprus from Europe and Central Asia, but not the USA (Fig. 3). HCV transmissions were exported to Belarus, Great Britain, and Uzbekistan (Core-E1), China, France, and Greece (NS5B). The viral mobility migration pattern is described in Supplementary Tables S1c and S2c.

The inferred viral mobility for the HCV epidemic in Cyprus suggests a diverse trans-continental pattern for each subtype, with infections originating from nearby and distant countries. 1a transmissions originated from limited locations in Western Europe, Southeastern Asia, and Oceania, 1b transmissions from Western and Eastern European areas, from Central and Eastern Asia, and Oceania, and 3a transmissions from Eastern and Western Europe, Central Asia, and Oceania. The USA provided a significant source for only one of the three subtypes (1b) analysed. Cyprus was a source for transmissions to several areas for subtypes 1b and 3a, but not for subtype 1a for which outgoing viral mobility was limited.

### Phylogenetic analysis – within country dispersal patterns

Due to incarcerated individuals and PWID being part of high-risk groups for HCV infection, the origin of HCV transmissions among these populations in Cyprus were specially analysed. Phylogenetic analysis was performed for these groups using sequences from different risk groups with a globally sampled dataset as a reference. These results were compared to the analysis of HCV infection within the general population (i.e. low risk group) in Cyprus. All analyses were only conducted for subtypes 1b and 3a which were almost exclusively the subtypes identified in these high-risk groups^13,14^.

**Figure 4:**
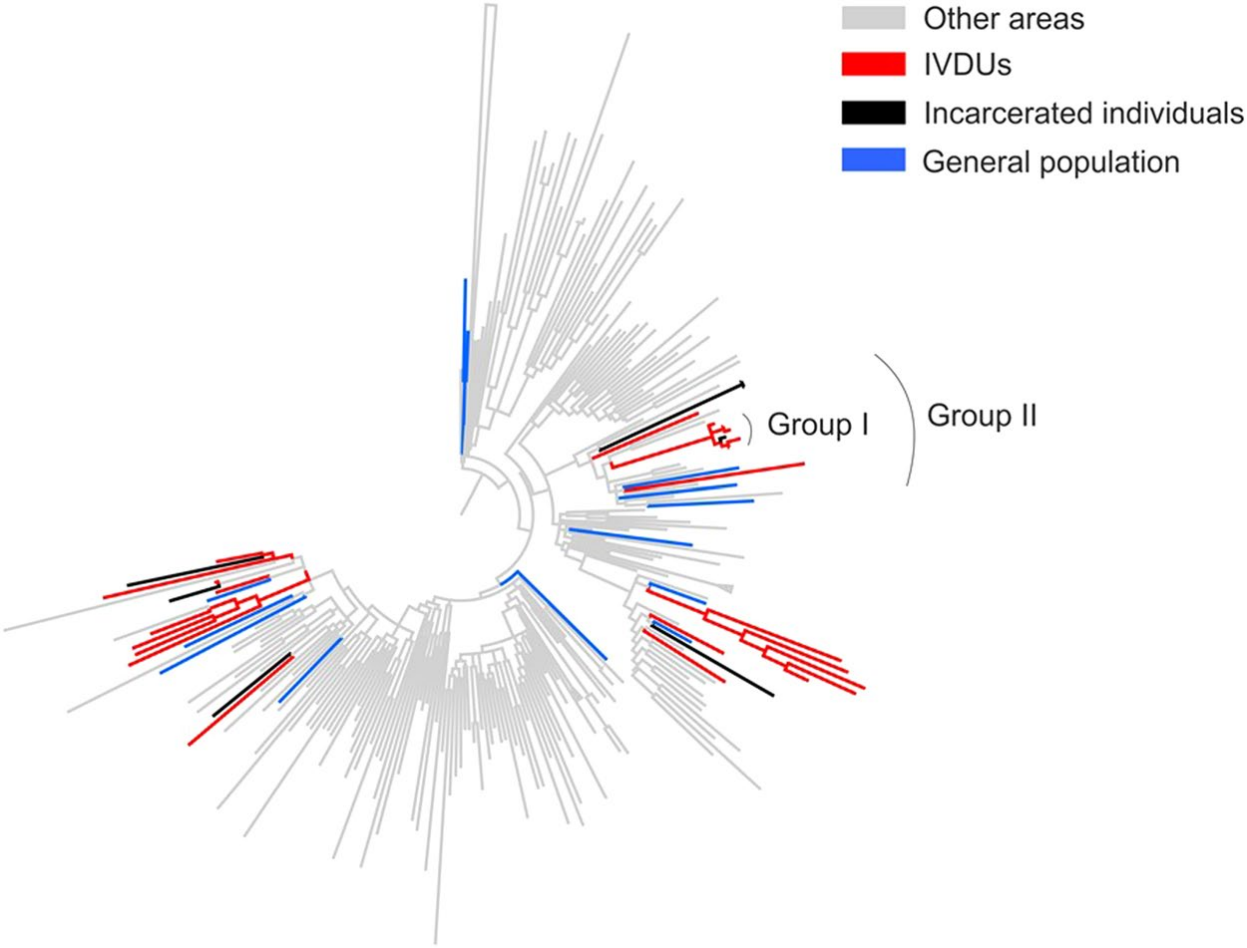
Phylogenetic tree. Phylogenetic tree using a globally sampled subtype 3a dataset and sequences from Cyprus. The transmission risk groups are shown in different colors.

Phylogenetic analysis for subtype 3a sequences in the Core-E1 region revealed that most HCV sequences from incarcerated individuals (5 out of 7, 71%) clustered with sequences from the general population and PWID from Cyprus (Fig. 4). Specifically, 4 of them (57%) grouped together with PWID sequences and 1 showed close phylogenetic relationship with single isolates from both the general population and PWID. While the exact origin of the latter sequence cannot be identified, a plausible hypothesis is that it originated from an PWID. A single case fell within a monophyletic group of five sequences from PWID in Cyprus (group I). The branch lengths of the whole group were very short suggesting a recent origin of HCV transmissions within the group.

The remaining two sequences from the incarcerated individuals clustered together with very short branches suggesting a recent common origin of infection. They fell within a monophyletic group of sequences (group II) sampled from Cyprus, Uzbekistan and Belarus. The majority of sequences from Cyprus within group II (7 out of 13, 54%) were from PWID; four were from the general population and two were from the target group. The remaining non-Cypriot sequences (collected from Uzbekistan) were also from PWID, while this specific demographic information from Belarus was not available. Given that the majority of strains within group II belonged to sequences from PWID, the most plausible hypothesis is that the putative origin of infection for the two incarcerated individuals was due to transmission from an PWID or drug paraphernalia. These findings were also confirmed in NS5B where identical phylogenetic clustering between sequences from incarcerated individuals and PWID from different areas was detected.

Similar analyses for subtype 1b (Core-E1), revealed that three out of four sequences from the target group (75%) clustered directly with PWID strains from Cyprus, although the putative origin of the last infection could not be identified. One sequence from incarcerated individuals fell within a cluster of three sequences from PWID connected with short branches. Phylogenetic analysis in NS5B confirmed only the clustering for the one sequence from among incarcerated individuals. This may be due to the larger number of references included in the NS5B region. Based on findings in Core-E1, the putative origin of most HCV transmissions among the incarcerated population is likely from PWID although from less evidence than for subtype 3a. While HCV sequences from incarcerated individuals sometimes group with the general population, the results in both the Core-E1 and NS5B regions suggest close phylogenetic relationships with PWID in subtypes 1b and 3a on an increasingly global scale.

## Discussion

This study used phylogeographic analysis to investigate the origins of HCV transmission to and from Cyprus and trace the global origin and dynamics of infections among incarcerated individuals in Cyprus. Among the observed HCV subtypes, phylogeographic analysis revealed a diverse pattern of viral mobility between Cyprus and other countries. In subtype 1b, which is the dominant clade among the general population, HCV transmissions to Cyprus originated from globally distributed countries in the Americas, Oceania, Southeastern Asia, and Europe. Cyprus was also a source for outgoing international 1b subtype transmissions across a broad geographical range. These observations suggest the bidirectional nature of the viral mobility of HCV subtype 1b in analyses of both the Core-E1 and NS5B region. Similar bidirectional viral mobility was indicated by analyses of the 3a subtype. However, analyses indicated that Cyprus was rarely a source of subtype 1a transmissions to other countries as it was a sink for viral transmission of this subtype. This finding is likely a result of the low prevalence of this subtype^11^.

Aggregately, the observed subtypes transmitted to Cyprus had origins in four continents, suggesting a complex transmission dynamic with trans-continental characteristics. HCV transmission occurred from the Americas and Western Europe, but also notably from Eastern Europe, Asia, and Oceania. These dynamics are like a result of Cyprus’ close ties with both Eastern and Western Europe in addition to its geographic location as a crossroads of three continents. No transmission to or from Africa was observed regarding to Cyprus despite their geographic proximity. While African countries have high prevalences of multiple HCV genotypes, these genotypes have demonstrated minimal transcontinental distribution and since African data is often not well documented and sequence data is likely not as readily available in online repositories^22,23^. However, the geographically widespread sequence data that is currently available is still useful for understanding the phylogeographic trends of HCV and elucidating the difference distribution and movement patterns among the subtypes.

Currently, the distribution of HCV clades (genotypes and subtypes) differs greatly across geographic areas and between transmission risk groups (e.g. PWID, blood transfusion recipients, the general population, etc.). This suggests that viral dissemination has followed distinct patterns across the globe and has been shaped by these specific factors^5^. In the current study, it was found that HCV dispersal in Cyprus follows complex subtype-specific transcontinental patterns, reinforcing the trend of risk factors being associated with subtype transmission.

Although modern transmission patterns are becoming better understood, it is not known when HCV developed within animal populations and how the disease spread to humans. It is likely that the genotypes of HCV arose in humans at different timeframes and have spread at different rates with varying geographic distributions^24^. The exponential growth of the epidemic in the human population began in the 1940s as a result of the widespread transfusion of blood and blood products^7^. Later the virus also began to spread more extensively due to intravenous drug use, drug trafficking, and human movement. Some specific transmission mechanisms are now associated with individual subtypes. The viral migration patterns of subtype 3a can likely be explained by the movement of drug trafficking and PWID, since the subtype primarily circulates among PWID^13^. While the transmission of subtypes 1b, 2a, and 2b are often linked to iatrogenic causes^25^, the dynamics driving movement of subtypes 1a and 1b within the scope of this study are more difficult to account for and merit further work on intra- and transnational transmission.

This study is one among few to trace the viral mobility into and out of a specific country using a global reference dataset^7,26^. In Scotland, it was shown that HCV subtype 1a and 3a sequences formed distinct clusters located between reference sequences from different sampled areas. Similarly, subtype 1a and subtype 1b sequences from Greece were dispersed across a globally constructed tree^7^ and levels of transmission networking among PWID were higher for subtype 3a than 1a^27^. Although few studies of this sort have been conducted, trends of HCV dissemination generally follow more distinct patterns than the HIV-1 pandemic, where most transmissions in the Western world were due to a single clade (subtype B), which originated in the Caribbean^20,28^. The greater complexity of the dissemination of HCV than of HIV-1 is likely due to differences in the epidemiology of the pathogens. HCV has infected humans for a longer time and over a wider geographic distribution prior to its exponential growth than HIV-1. Different transmission routes and factors related to the host response in humans likely also shape how the viruses have distinctly spread. The results of this study suggest that subtype-specific HCV transmission patterns exist within not only within Cyprus but also between Cyprus and widely globally distributed countries. Additional studies are needed to better understand the spatial characteristics and factors that have and will shape the global HCV epidemic.

While this study examined a broad group of individuals with HCV, incarcerated individuals and PWID were populations of note, given the high-risk of HCV among them. While phylogeographic analysis was used to trace transnational patterns of transmission, it also revealed the dispersal patterns among these populations in Cyprus. Within the incarcerated population, newest infections arose from PWID already within Cyprus. These findings indicate a shared subtype within these populations, suggesting how certain risk factors can predispose to infection by a specific subtype. Highlighting this finding, two incarcerated individuals displayed evidence of a common source of infection within a cluster of PWID from Cyprus and other areas, reinforcing trends between countries and risk groups. These results were robust across a variety of genotypes. This suggests that injections of intravenous drugs pose a risk for HCV transmission among incarcerated individuals. Responding to these transmission trends, HCV prevention should be intensified and target this high-risk group with risk-appropriate measures such as volunteer testing for HCV and treatment using the direct acting antivirals (DAAs) against HCV.

Using state of the art phylogeographic methods, this study analysed the movement and dissemination of the major HCV subtypes in Cyprus (1a, 1b, and 3a) to answer two questions: what are the global geographic patterns of HCV transmission into and out of Cyprus and what are the patterns of transmission among the general and high-risk populations within Cyprus? To frame the both elements of the work within a maximally comprehensive global context, the analyses included all sequences available on public databases as references. Given the large number of sequences analyzed, statistical phylogeography was used due to the long computation time required for Bayesian methods. One of the potential limitations in this study is the lack of available HCV sequences from many geographic locations. To improve the analytical capacity of the existing data, inferences were performed in 2 different partial genomic regions (Core-E1 and NS5B) and merged migration events from both regions. This study highlights the complexity of HCV dissemination within, into, and out of Cyprus. While the movement of HCV does not follow a specific pattern like the HIV-1 epidemic, Cyprus, as an island at the geographic crossroads of three continents, is a powerful place to begin unraveling the intricacies of the patterns of global HCV transmission. This complexity likely reflects the different routes for HCV epidemic transmission and dispersal, including blood and blood product transfusions, iatrogenic transmissions, and drug injections. Because these transmission mechanisms are often associated with different subtypes, phylogeographic analysis is a powerful tool for mapping how and where HCV subtypes are transmitted. As more sequences are made available from wider geographic regions, phylogeographic analysis will more effectively describe the complex patterns of the dissemination of HCV and other viral diseases so more informed approaches can be made to combatting these epidemics.

## Supplementary information

Supplementary Figures and Tables
