# Supplementary material for: HCV Phylogeography of the General Population and High-Risk Groups in Cyprus Identifies the Island as a Global Sink for and Source of Infection
Source: Sci Rep. 2019 Jul 11;9:10077. doi: 10.1038/s41598-019-46552-7 (PMC6624375; doi:10.1038/s41598-019-46552-7)
Supplement: Supplementary file 1 — Supplementary Figures and Tables [file 41598_2019_46552_MOESM1_ESM.pdf]

HCV Phylogeography of the General Population and High-Risk Groups in Cyprus Identifies the Island as a Global Sink for and Source of Infection

Dimitrios Paraskevis[1,+], Dora C. Stylianou[2,+], Johana Hezka[2], Zachariah Stern[2], Martha Oikonomopoulou[1], Ioannis Mamais[1], Cyprus PWID Network and Leondios G. Kostrikis[1,*]

[1]Department of Hygiene, Epidemiology and Medical Statistics, Medical School, National and Kapodistrian University of Athens, Athens Greece
[2]Department of Biological Sciences, University of Cyprus, Nicosia, Cyprus

[+] these authors contributed equally to this work,
[*] lkostrik@ucy.ac.cy

Supplementary Figures and Tables

Supplementary Figure S1A

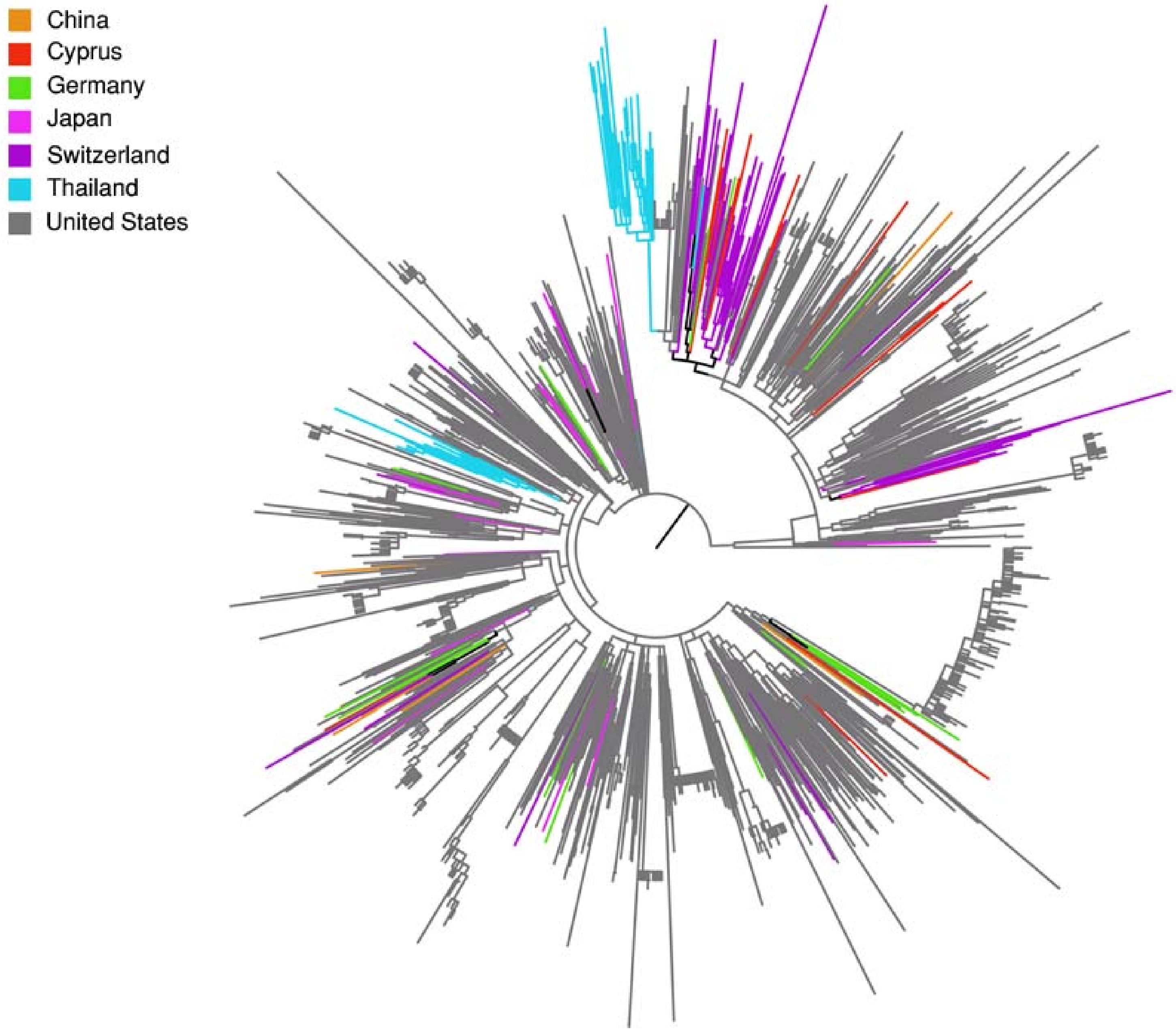

Supplementary Figure S1B

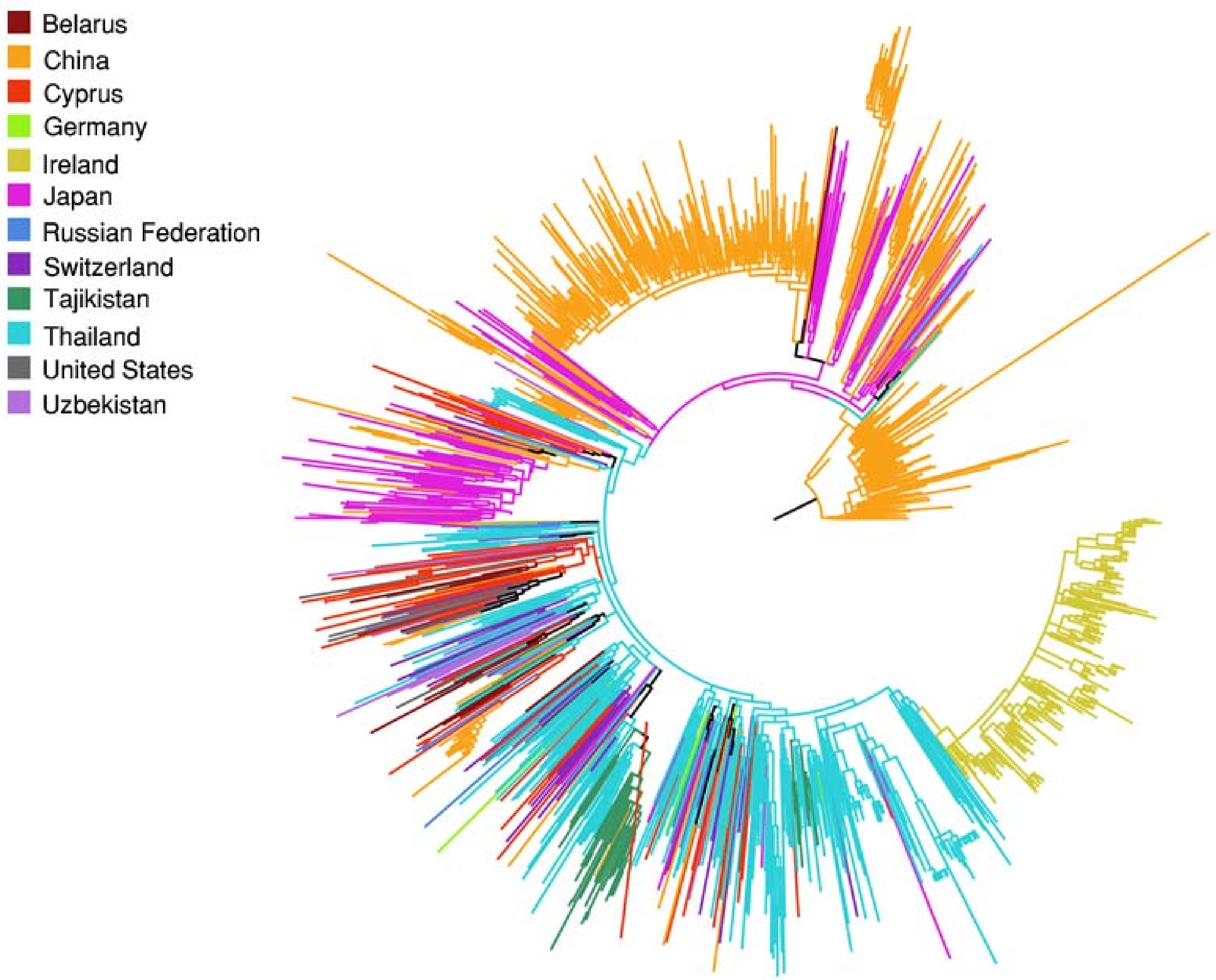

Supplementary Figure S1C

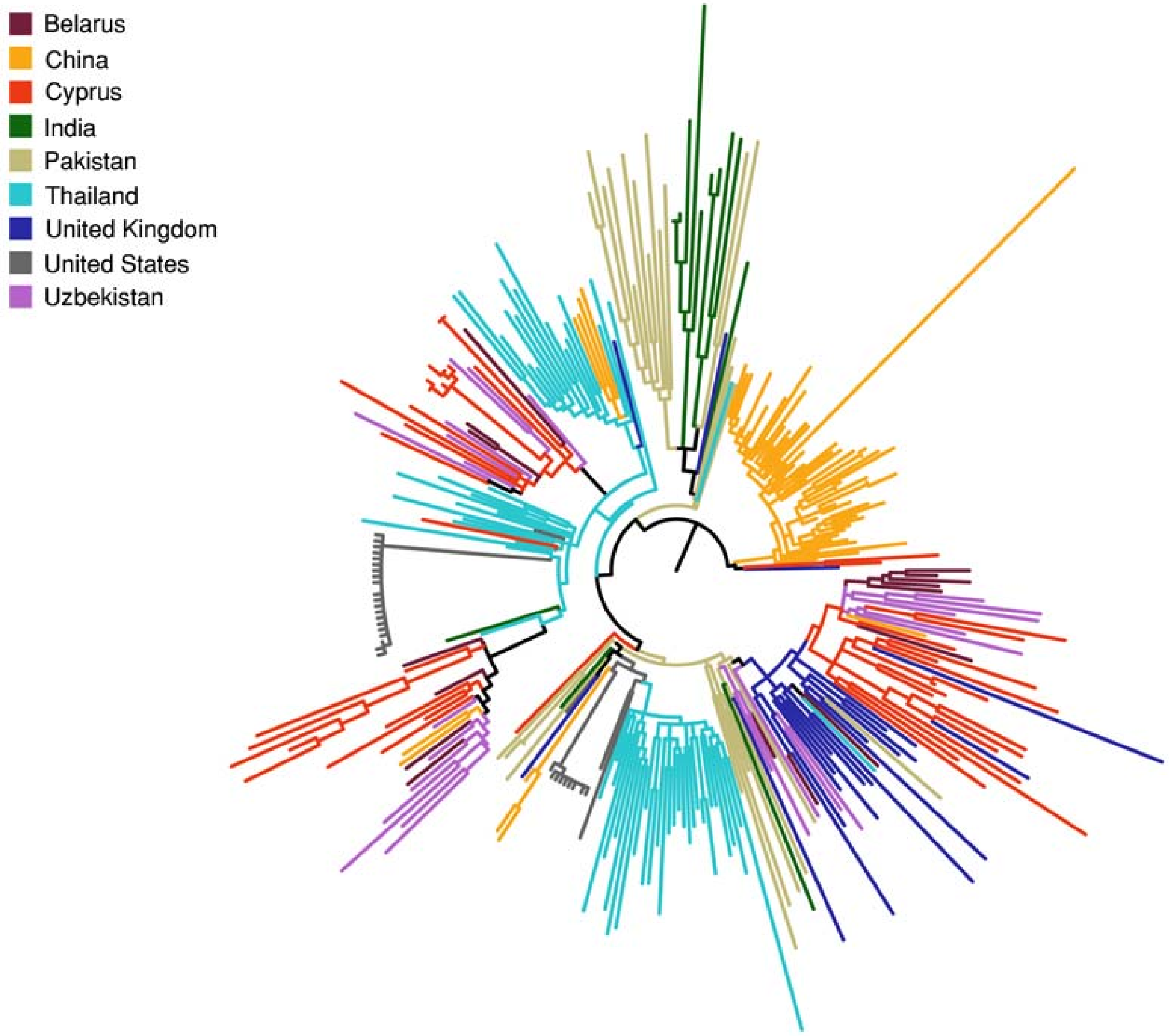

Supplementary Figure S1. **Phylogeographic trees of Core-E1**. Maximum likelihood (ML), midpoint-rooted phylogeographic tree showing HCV viral clades in different colors by country. **(A)** Core-E1 subtype 1a, **(B)** Core-E1 subtype 1b, and **(C)** Core-E1 subtype 3a.

Supplementary Figure S2A

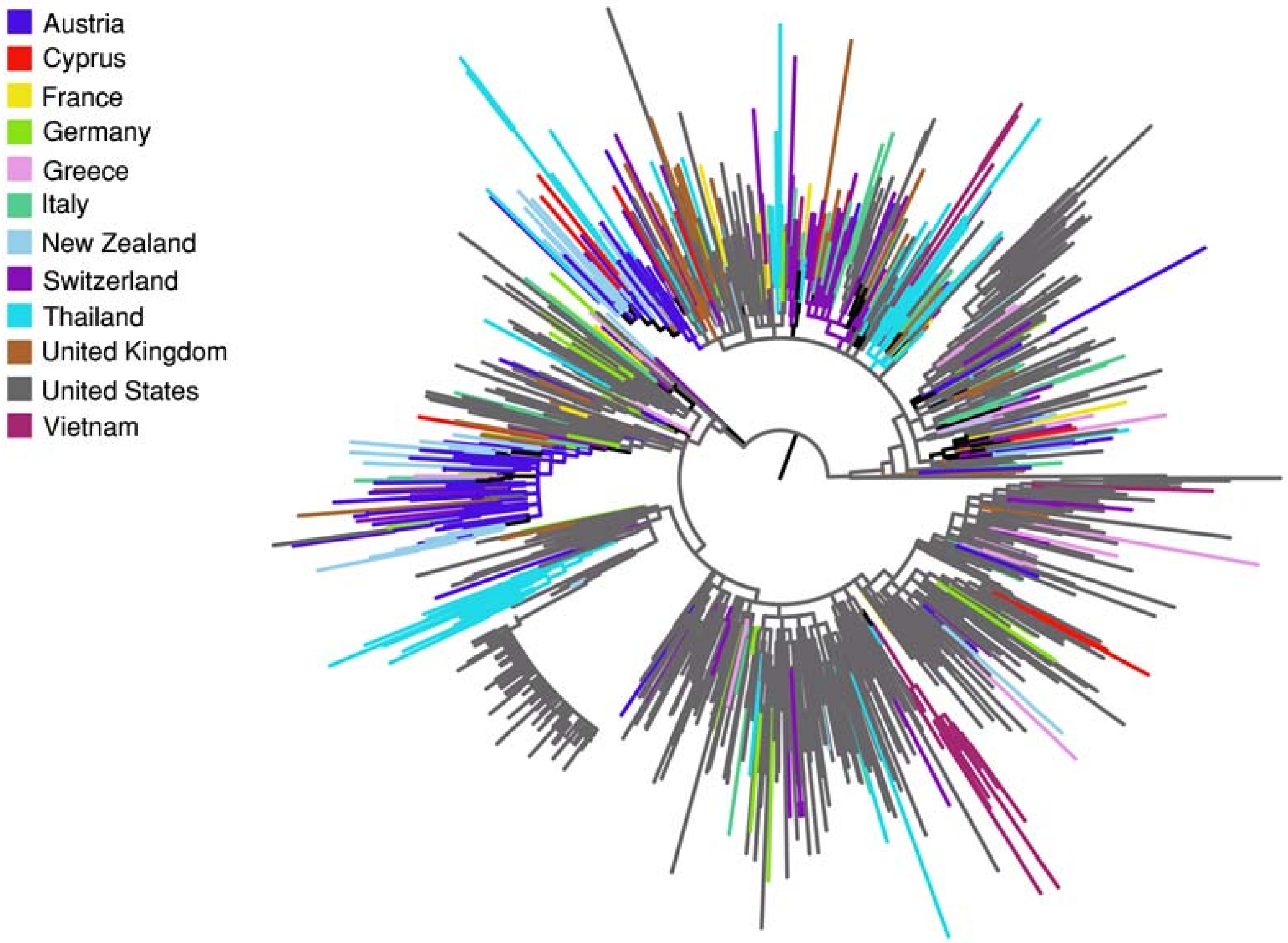

Supplementary Figure S2B

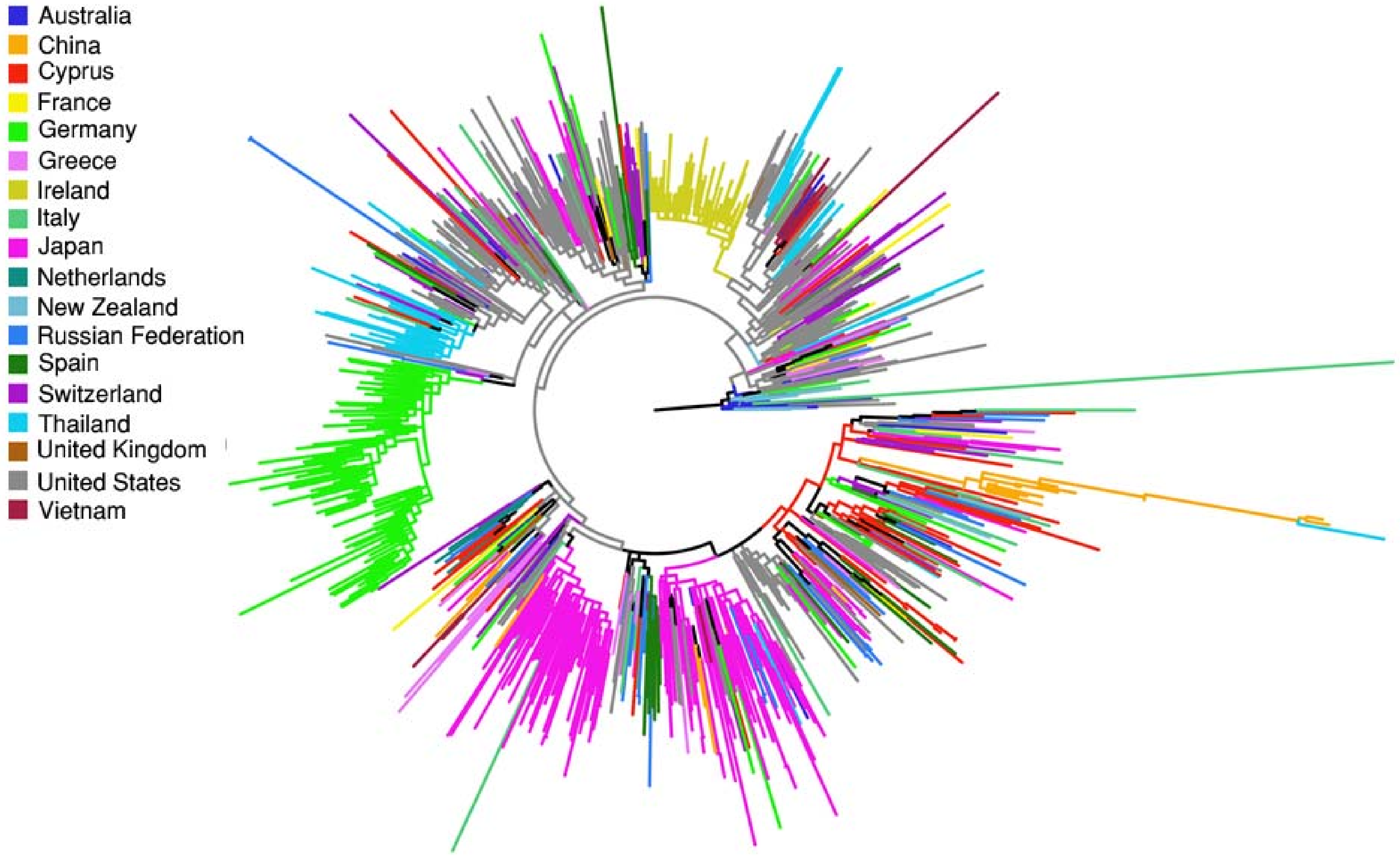

Supplementary Figure S2C

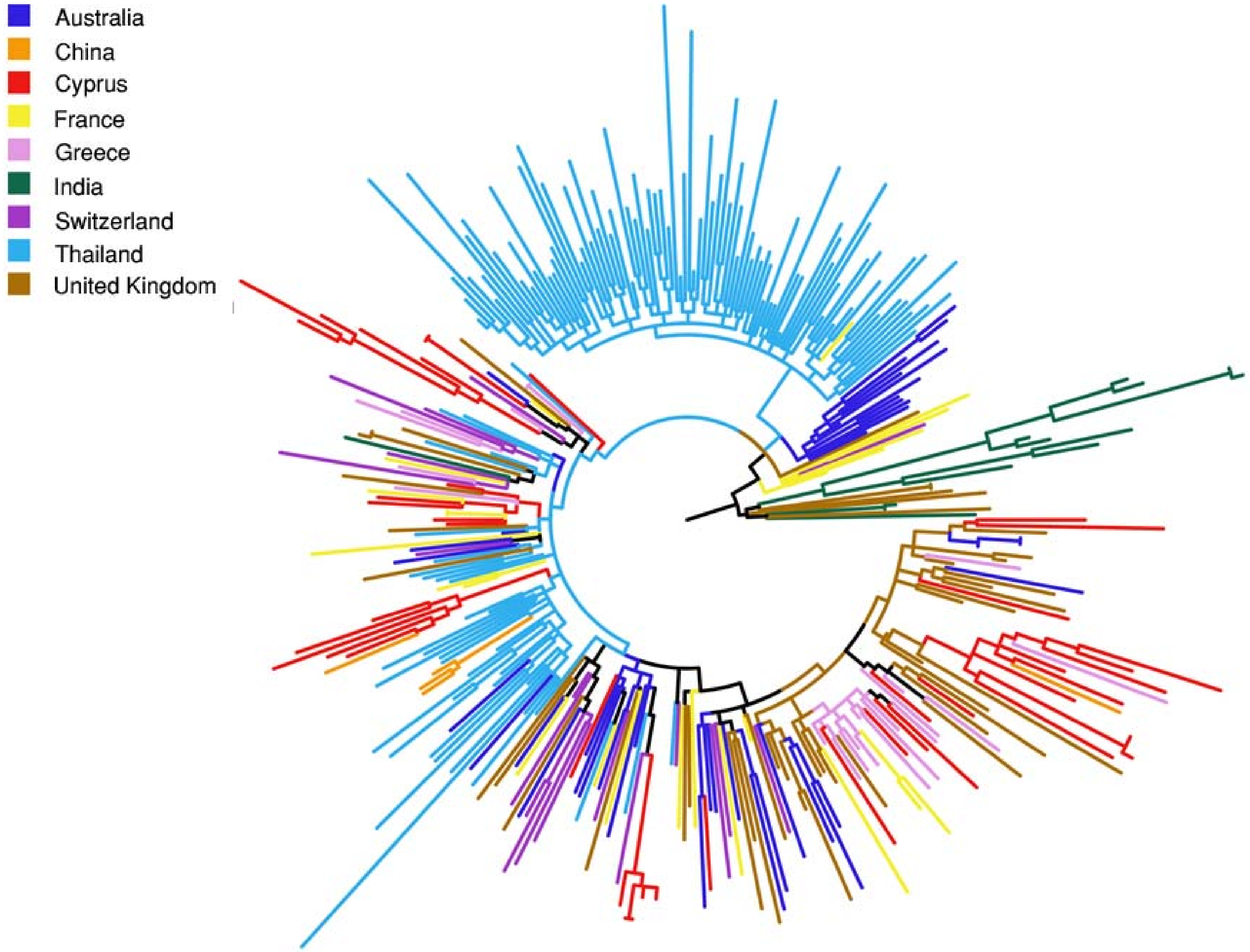

Supplementary Figure S2. **Phylogeographic trees of NS5B**. Maximum likelihood (ML), midpoint-rooted phylogeographic tree showing HCV viral clades in different colors by country. **(A)** NS5B subtype 1a, **(B)** NS5B subtype 1b, and **(C)** NS5B subtype 3a.

Supplementary Table S1. Core-E1 Viral Mobility. Means of observed migration events across all bootstrap trees between different countries. Cells in bold indicate statistically significant pathways (compared to the null- hypothesis of panmixis) after Bonferroni correction for multiple comparisons. Core -E1 subtype **(A)** 1a, **(B)** 1b, and **(C)** 3a.

Supplementary Table S1a. Mean of observed migration events across all bootstrap trees for each pathway for HCV subtype 1a encoding Core-E1 region

	Importing to							
		**China**	**Cyprus**	**Germany**	**Japan**	**Switzerland**	**Thailand**	**USA**
Exporting from	**China** (7)		0.017	0.010	0.000	**0.120**	0.017	0.000
	**Cyprus** (14)	0.000		**0.051**	0.000	**0.098**	0.015	**0.081**
	**Germany** (21)	0.015	0.015		0.005	**0.042**	0.005	**0.125**
	**Japan** (14)	0.000	0.000	0.005		0.000	0.000	0.000
	**Switzerland** (48)	0.015	**1.895**	**1.419**	0.034		**0.377**	**2.4**
	**Thailand** (55)	0.000	**0.059**	0.022	0.042	**0.081**		**0.632**
	**US** (1279)	3.850	6.662	12.017	12.777	13.748	4.909	

Note. Cells in bold indicate statistically significant pathways (compared to the null-hypothesis of pamixis) after Bonferroni correction for multiple comparisons. USA, United States of America. For each country, the number in parenthesis indicate the number of HCV subtype 1a sequences (encoding the CORE-E1 region) isolated from and used in the analysis.

Supplementary Table S1b. Mean of observed migration events across all bootstrap trees for each pathway for HCV subtype 1b encoding the Core-E1 region

	Importing to												
		**Belarus**	**China**	**Cyprus**	**Germany**	**Ireland**	**Japan**	**Russia**	**Switzerland**	**Tajikistan**	**Thailand**	**USA**	**Uzbekistan**
	**Belarus** (16)		**0.081**	**0.252**	0.000	0.000	0.042	0.012	0.032	**0.034**	0.000	**0.360**	**0.279**
Exporting from	**China** (529)	0.208		0.975	0.044	0.000	5.51	0.262	0.770	0.659	0.091	2.306	0.397
	**Cyprus** (53)	**0.985**	**0.760**		**0.071**	0.000	**0.282**	**0.223**	**1.189**	**0.951**	0.017	0.380	**0.826**
	**Germany** (7)	0.000	0.000	0.000		0.000	0.000	0.000	0.002	0.005	0.000	0.000	0.000
	**Ireland** (192)	0.000	0.015	0.235	0.000		0.005	0.000	0.007	0.000	0.012	0.265	0.000
	**Japan** (122)	0.223	**10.025**	0.635	0.022	0.000		0.076	**0.652**	**0.409**	0.049	**1.892**	0.137
	**Russia** (7)	0.005	0.015	**0.037**	0.000	0.000	0.002		0.002	0.002	0.015	0.005	0.005
	**Switzerland** (40)	**0.074**	**0.377**	**0.615**	**0.071**	0.005	**0.218**	**0.056**		**0.093**	0.049	**0.488**	**0.118**
	**Tajikistan** (13)	**0.039**	**0.100**	**0.059**	0.015	0.000	0.032	0.005	**0.044**		0.002	0.034	**0.056**
	**Thailand** (39)	0.002	**1.002**	0.047	0.007	0.000	0.049	0.015	**0.100**	0.015		**0.588**	0.002
	**US** (274)	**3.407**	7.221	**13.243**	**3.887**	0.902	**8.88**	**2.098**	**15.385**	**2.588**	**5.990**		**3.358**
	**Uzbekistan** (12)	**0.091**	0.029	**0.284**	0.000	0.000	0.012	0.012	**0.078**	**0.159**	0.002	0.017	

Note. Cells in bold indicate statistically significant pathways (compared to the null-hypothesis of pamixis) after Bonferroni correction for multiple comparisons. USA, United States of America. For each country, the number in parenthesis indicate the number of HCV subtype 1b sequences (encoding the CORE-E1 region) isolated from and used in the analysis.

Supplementary Table S1c. Mean of observed migration events across all bootstrap trees for each pathway for HCV subtype 3a encoding Core-E1 region

	Importing to									
		**Belarus**	**China**	**Cyprus**	**India**	**Pakistan**	**Thailand**	**GB**	**USA**	**Uzbekistan**
Exporting from	**Belarus** (16)		0.147	**0.526**	0.009	0.026	0.022	**0.289**	0.009	**0.458**
	**China** (70)	0.018		0.072	0.031	0.211	0.175	0.105	0.009	0.048
	**Cyprus** (44)	**1.487**	0.377		0.031	0.079	0.121	**1.423**	0.057	**1.873**
	**India** (24)	0.004	0.026	0.011		0.169	0.046	0.116	0.044	0.050
	**Pakistan** (28)	0.208	0.401	**0.590**	**1.583**		**0.91**	**1.175**	**0.511**	**0.649**
	**Thailand** (65)	0.107	1.632	1.379	0.919	0.941		0.787	1.728	0.417
	**GB** (22)	**1.825**	0.265	**1.682**	**0.257**	**0.866**	**1.026**		0.204	**1.173**
	**US** (43)	0.018	0.079	0.046	0.081	0.057	0.246	0.046		0.094
	**Uzbekistan** (25)	**1.875**	**0.770**	**2.270**	0.053	0.169	0.057	0.294	0.061	

Note. Cells in bold indicate statistically significant pathways (compared to the null-hypothesis of pamixis) after Bonferroni correction for multiple comparisons. GB, United Kingdom; USA, United States of America. For each country, the number in parenthesis indicate the number of HCV subtype 3a sequences (encoding the CORE-E1 region) isolated from and used in the analysis.

Supplementary Table S2. **NS5B Viral Mobility**. Means of observed migration events across all bootstrap trees between different countries. Cells in bold indicate statistically significant pathways (compared to the null- hypothesis of panmixis) after Bonferroni correction for multiple comparisons. NS5B subtype **(A)** 1a, **(B)** 1b, and **(C)** 3a.

Supplementary Table S2a. Mean of observed migration events across all bootstrap trees for each pathway for HCV subtype 1a encoding NS5B region

	Importing to												
		**Australia**	**Cyprus**	**France**	**Germany**	**Greece**	**Italy**	**New Zealand**	**Switzerland**	**Thailand**	**GB**	**USA**	**Vietnam**
	**Australia** (71)		**0.557**	**0.183**	**0.863**	**0.437**	**0.733**	**7.173**	**2.343**	**1.087**	**2.013**	**1.683**	0.027
Exporting from	**Cyprus** (11)	0.017		0.000	0.000	0.003	0.007	0.03	0.017	0.017	0.000	0.020	0.007
	**France** (15)	**0.160**	0.003		**0.057**	**0.090**	0.027	0.000	**0.043**	0.050	0.010	**0.107**	0.000
	**Germany** (32)	**0.140**	0.010	0.007		0.037	0.030	0.027	0.087	0.043	0.043	**0.233**	0.013
	**Greece** (24)	0.027	0.020	0.043	**0.110**		0.037	0.01	0.043	0.010	0.057	0.043	0.000
	**Italy** (32)	**0.230**	**0.043**	**0.127**	**0.213**	**0.203**		**0.063**	**0.643**	**0.147**	**0.397**	**0.283**	0.040
	**New Zealand** (49)	**3.470**	**0.430**	0.037	0.110	**0.117**	**0.200**		**0.370**	**0.837**	**0.613**	**0.443**	0.003
	**Switzerland** (80)	**0.923**	**0.873**	**0.687**	**1.337**	**1.367**	**2.947**	**0.317**		**0.873**	**1.233**	**2.213**	**0.323**
	**Thailand** (70)	**0.627**	**0.253**	**0.460**	0.067	**0.230**	**0.527**	**0.31**	**0.830**		**1.090**	**1.833**	**0.753**
	**GB** (42)	**0.297**	**0.150**	**0.137**	**0.103**	**0.210**	**0.177**	**0.200**	**0.797**	**0.370**		**0.673**	0.053
	**US** (629)	14.58	3.627	6.060	16.93	11.843	9.033	9.643	23.293	17.22	13.157		3.613
	**Vietnam** (21)	0.003	0.003	0.000	0.000	0.000	0.000	0.000	0.003	0.063	0.000	0.047	

Note. Cells in bold indicate statistically significant pathways (compared to the null-hypothesis of pamixis) after Bonferroni correction for multiple comparisons. GB, United Kingdom; and USA, United States of America. For each country, the number in parenthesis indicate the number of HCV subtype 1a sequences (encoding the NS5B region) isolated from and used in the analysis.

	Importing to																	
		**AU**	**CN**	**CY**	**FR**	**DE**	**GR**	**IE**	**IT**	**JP**	**NL**	**NZ**	**RU**	**ES**	**CH**	**TH**	**GB**	**USA**	**VN**
	**AU** (26)		0.006	0.086	0.039	0.208	0.064	0.000	**0.161**	0.094	0.006	**0.908**	0.139	0.05	**0.153**	**0.183**	0.008	**0.422**	0.000
Importing from	**CN** (23)	0.000		0.025	0.014	0.036	0.017	0.000	0.022	**0.267**	0.000	0.000	0.039	0.003	0.039	**0.783**	0.000	0.081	0.05
	**CY** (54)	**0.575**	0.05		**0.150**	0.569	**0.383**	0.006	**1.408**	0.742	0.114	**0.558**	**1.078**	**1.600**	**0.65**	0.189	**0.144**	**0.661**	0.036
	**FR** (12)	0.017	0.008	**0.067**		0.014	0.006	0.003	0.017	0.031	0.006	0.003	0.008	0.028	0.017	0.008	0.006	0.006	0.003
	**DE** (148)	0.053	0.019	0.336	0.058		0.142	0.003	0.289	0.131	0.003	0.017	0.128	0.092	0.456	0.144	0.025	0.711	0.014
	**GR** (22)	0.033	0.003	**0.233**	0.042	0.083		0.000	**0.108**	0.094	0.006	0.006	**0.161**	**0.097**	0.086	0.006	0.019	0.083	0.014
	**IE** (72)	0.011	0.011	0.028	0.008	0.000	0.028		0.022	0.264	0.000	0.000	0.025	0.028	0.081	0.019	0.000	0.742	0.061
	**IT** (30)	0.069	0.028	**0.358**	**0.192**	0.256	**0.144**	0.000		**0.278**	0.011	0.081	**0.214**	0.075	**0.203**	0.022	0.014	0.164	0.000
	**JP** (153)	1.825	**2.753**	1.714	0.35	1.131	0.783	0.831	2.056		0.033	0.083	1.725	1.061	1.306	0.842	0.183	4.031	0.444
	**NL** (6)	**0.036**	0.000	**0.536**	0.006	0.000	0.008	0.000	0.014	0.006		0.000	**0.714**	0.003	**0.064**	0.000	0.000	0.008	0.000
	**NZ** (13)	**0.622**	0.003	**0.094**	0.053	0.036	0.022	0.000	**0.131**	0.053	0.003		0.039	0.022	0.058	**0.269**	0.008	**0.233**	0.000
	**RU** (38)	0.061	0.036	**0.744**	0.075	0.253	**0.322**	0.003	**0.336**	0.164	**0.178**	0.058		0.122	**0.506**	0.039	0.022	0.208	0.011
	**ES** (33)	0.131	0.022	**0.789**	**0.267**	0.094	0.131	0.003	**0.483**	0.142	0.017	0.006	**0.300**		**0.769**	0.022	0.033	**0.433**	0.000
	**CH** (49)	0.142	0.028	**0.411**	**0.233**	**0.556**	0.192	0.014	**0.311**	0.233	0.022	0.067	0.253	**0.528**		0.064	0.083	0.464	0.014
	**TH** (48)	0.008	0.003	0.331	0.006	**0.553**	0.036	0.003	**0.575**	0.044	0.000	0.042	0.019	0.044	**1.186**		0.056	**1.783**	0.011
	**GB** (7)	0.003	0.000	0.003	0.000	0.000	0.000	0.000	0.000	0.000	0.000	0.000	0.000	0.000	0.003	0.000		0.000	0.000
	**US** (231)	**7.569**	0.767	10.011	**4.358**	9.111	**7.572**	1.053	6.961	11.381	0.744	2.328	7.831	8.081	13.317	5.408	**3.500**		1.728
	**VN** (12)	**0.489**	0.000	**0.181**	0.006	0.000	0.003	0.003	0.006	0.014	0.000	0.000	0.003	0.003	0.000	0.025	0.000	**0.492**	

Supplementary Table S2b. Mean of observed migration events across all bootstrap trees for each pathway for HCV subtype 1b encoding NS5B region

Note. Cells in bold indicate statistically significant pathways (compared to the null-hypothesis of pamixis) after Bonferroni correction for multiple comparisons. AU, Australia; CN, China; CY, Cyprus; FR, France; DE, Germany; GR, Greece; IE, Ireland; IT, Italy; JP, Japan; NL, Netherlands; RU, Russian Federation; ES, Spain; CH, Switzerland; TH, Thailand; GB, United Kingdom; USA, United States of America and VN, Vietnam. For each country, the number in parenthesis indicate the number of HCV subtype 1b sequences (encoding the NS5B region) isolated from and used in the analysis.

Supplementary Table S2c. Mean of observed migration events across all bootstrap trees for each pathway for HCV subtype 3a encoding NS5B region

	Importing to									
		**Australia**	**China**	**Cyprus**	**France**	**Greece**	**India**	**Switzerland**	**Thailand**	**GB**
Exporting from	**Australia** (42)		0.081	**1.103**	**1.175**	0.431	0.314	**0.722**	**1.283**	**1.253**
	**China** (5)	0.000		0.000	0.000	0.000	0.000	0.003	0.003	0.006
	**Cyprus** (45)	0.475	**1.831**		**0.972**	**1.197**	0.222	0.478	0.328	0.650
	**France** (25)	**0.458**	0.008	0.325		0.167	0.053	**0.350**	0.222	**0.497**
	**Greece** (22)	**0.275**	0.003	**2.383**	**0.986**		0.006	**0.192**	0.042	**1.214**
	**India** (15)	0.008	0.000	0.008	0.019	0.006		0.022	0.039	0.119
	**Switzerland** (19)	**0.647**	0.017	**0.325**	**0.514**	**0.642**	0.119		**0.325**	**0.608**
	**Thailand** (130)	4.319	0.461	1.292	3.139	0.667	0.653	2.206		2.447
	**GB** (58)	**5.083**	0.053	**2.756**	**2.661**	**2.425**	**0.867**	**1.636**	0.842	

Note. Cells in bold indicate statistically significant pathways (compared to the null-hypothesis of pamixis) after Bonferroni correction for multiple comparisons. GB, United Kingdom. For each country, the number in parenthesis indicate the number of HCV subtype 3a sequences (encoding the NS5B region) isolated from and used in the analysis.
